# Colouterine Fistula as a Rare Cause of Postmenopausal Abnormal Uterine Bleeding: A Case Report

**DOI:** 10.3390/jcm13030783

**Published:** 2024-01-29

**Authors:** Cátia Silva, Cristina Monteiro, Fernando Barbosa, Inês Cunha, Rosália Coutada, Agostinho Carvalho

**Affiliations:** 1 Gynecological Department, Coimbra Hospital and University Centre, 3000-075 Coimbra, Portugal; 2 General Surgery Department, Local Health Unit of Alto Minho, 4904-858 Viana do Castelo, Portugal; cristina.monteiro@ulsam.min-saude.pt (C.M.); fernando.barbosa@ulsam.min-saude.pt (F.B.); 3 Gastroenterology Department, Local Health Unit of Alto Minho, 4904-858 Viana do Castelo, Portugal; ines.fonseca.cunha@ulsam.min-saude.pt; 4 Gynecological Department, Local Health Unit of Alto Minho, 4904-858 Viana do Castelo, Portugal; rosalia.coutada@ulsam.min-saude.pt (R.C.); agostinho.carvalho@ulsam.min-saude.pt (A.C.)

**Keywords:** abnormal uterine bleeding, menopause, colouterine fistula, diverticular fistula, colon diverticulosis, diverticulitis

## Abstract

Postmenopausal abnormal uterine bleeding is a common clinical problem addressed in gynaecological practice and should prompt clinical investigation due to the significant prevalence of malignant and premalignant lesions of the endometrium in this age group. Nevertheless, other causes should be considered, since its diagnostic and therapeutic management may differ considerably. Here, we present a case of a colouterine fistula due to chronic diverticulitis presenting with postmenopausal abnormal uterine bleeding. This is an infrequent occurrence and is caused by the rupture of a diverticular abscess into the uterine wall, resulting in an inflammatory adhesion of the colon and uterus, with necrosis and subsequent fistula formation. The clinical presentation is variable and may include abdominal pain, gastrointestinal tract symptoms, vaginal discharge, and abnormal uterine bleeding. The laboratory and imaging techniques may be not completely conclusive and definitive diagnosis can be made intraoperatively. There are different treatment options, with en bloc resection and primary anastomosis being used most often, allowing complete treatment. The prognosis for a colouterine fistula secondary to diverticulitis is excellent after surgery. This case highlights the importance of clinical suspicion of an unusual cause of uterine bleeding and an effective and multidisciplinary approach that allowed complete surgical treatment and patient recovery.

## 1. Introduction

Postmenopausal abnormal uterine bleeding (AUB) is a common clinical problem and occurs in approximately 4 to 11 percent of postmenopausal women [1]. This symptom requires a prompt clinical and aetiological evaluation, mainly to exclude malignant or premalignant lesions of the endometrium. It should include a detailed medical history, physical examination and diagnostic imaging, and laboratory tests that can help clarify clinical suspicions [2].

Colonic diverticular disease is a common disease in Western countries and, due to inflammation of the diverticula, may encompass complications such as haemorrhage, abscess formation, colonic perforation, and fistulisation to adjacent organs. Concerning complications with fistulisation, the most common organs affected are the urinary bladder and vagina [3]. Fistulisation between the colon and uterus due to diverticular disease is very unusual and few cases are reported in the literature.

A colouterine fistula due to diverticular disease is rare. In fact, colouterine fistula from other causes are more common and can happen in the sequence of spontaneous rupture of a gravid uterus, obstetric trauma, and pelvic malignancy and radiation [4].

Colouterine fistula can cause abnormal uterine bleeding due to chronic inflammation in the uterine wall adjacent to the colonic diverticulum. CT scans are routinely performed for suspected diagnosis but usually cannot provide a definitive diagnosis alone, which enhances the need for a proper clinical medical history, correlation with physical examination, and radiological and laboratory findings to select appropriate management. Resection is the treatment of choice for colouterine fistulas and, in most cases, en bloc sigmoid and uterus resection has now become the preferred technique, especially in cases where malignancy is suspected. This approach allows complete treatment and an excellent prognosis afterwards. 

We describe a patient with a history of AUB due to a colouterine fistula after chronic diverticulitis.

## 2. Case Presentation

This is a case of a seventy-one-year-old female, with a prior medical history of high blood pressure taking an oral hypertensive daily, without previous surgeries or other significant medical problems. She had two normal pregnancies and two eutocic deliveries; menopause occurred around fifty-one years of age, and she did not take any hormonal replacement therapy nor had prior history of gynaecological problems. 

**Figure 1:**
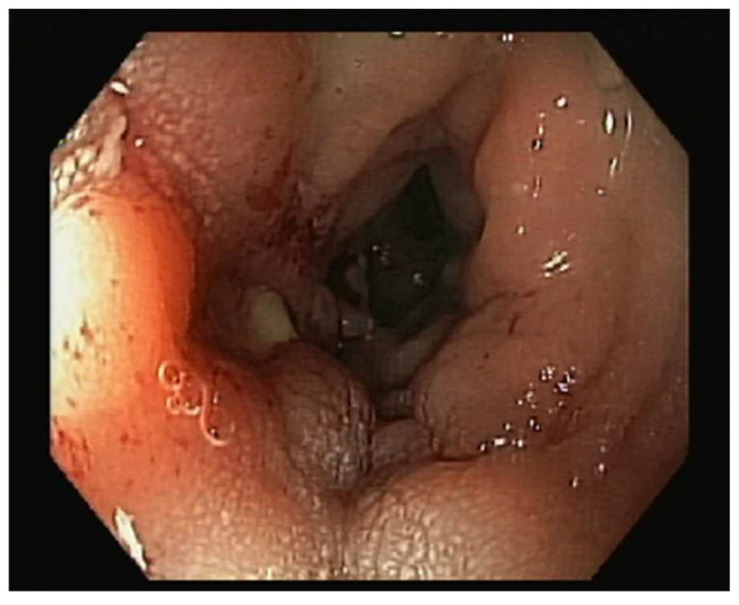

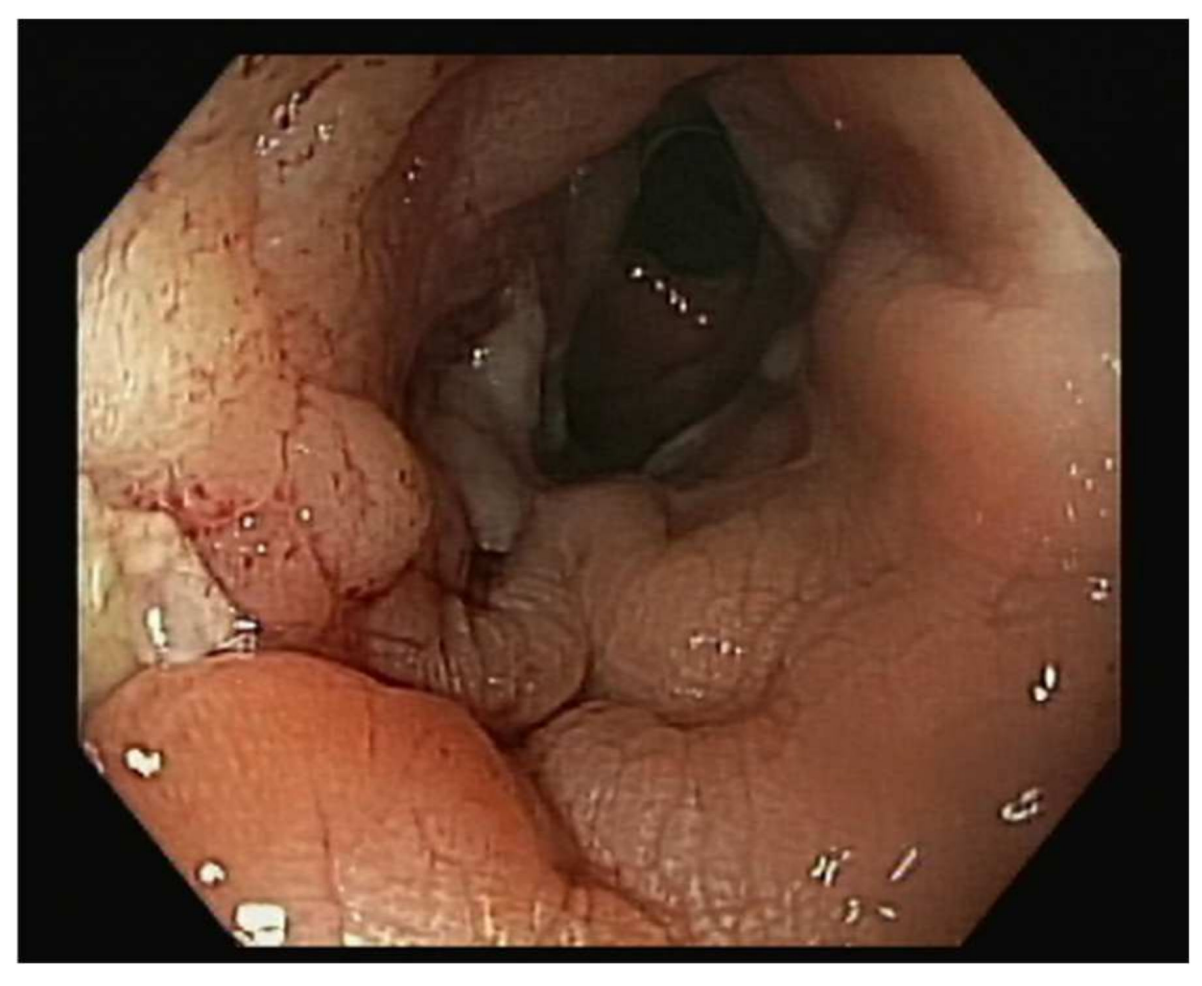
Inflammatory changes in sigmoid colon mucosa wall around 20 cm from anal verge, with a granular appearance in a significant part of the mucosal circumference. There was a suspicion of a fistulous tract with purulent drainage that was biopsied.

The patient was referred to the gastroenterology practice due to a routine faecal occult blood test that was positive and abnormal findings in the colonoscopy. She mentioned she had experienced vaginal bleeding in scarce quantities a few months prior, without other symptoms, namely changes in gastrointestinal habits, abdominal pain, bloating, haematochezia, weight loss, fever, or fatigue. The colonoscopy revealed inflammatory changes with a granular appearance in a significant part of the mucosal circumference in the sigmoid colon mucosa wall, approximately around 20 cm from the anal verge. There was a suspicion of a fistulous tract with purulent drainage (Figure 1) and that area was biopsied. The histopathological analysis of the biopsied areas showed chronic inflammatory unspecific colitis without malignant or premalignant changes and, therefore, a hypothesis of colonic ulcer or fistula was made. 

Due to abnormal vaginal bleeding, she was referred to the gynaecology practice. She denied other gynaecological or urinary tract symptoms and mentioned the bleeding was painless, as a light spotting, happening almost daily and having started a few months prior. On examination, she had normal vital signs, without fever. She was obese (body mass index of 32 kg/m^2^); an examination of the abdomen was unremarkable, without masses or pain with palpation. On speculum examination, she had vulvar and vaginal atrophy, the cervix had no changes, and no uterine or vaginal bleeding was noted. The vaginal discharge was normal and odourless. The uterus was felt to be of normal size for her age, movable with bimanual palpation, and the adnexa were not palpable.

The transvaginal ultrasound showed the uterus in anteverted position with three small FIGO type 3–4 myomas, all less than 1cm in maximum diameter, regular outer contour, and an endometrium thickness of 3 mm, with regular lining. The adnexa had normal appearance and a small amount of sonolucent fluid in the rectouterine pouch was noted. The patient did not refer to tenderness or pain during the physical examination and ultrasound exam.

The diagnostic hysteroscopy through vaginoscopy showed an atrophic endometrium without intracavitary lesions; both tubal orifices were seen and there was no evidence of visible fistulous tracts or other abnormal drainage or liquid in the uterine cavity. The sampled endometrium did not find endometrium malignant or premalignant changes.

**Figure 2:**
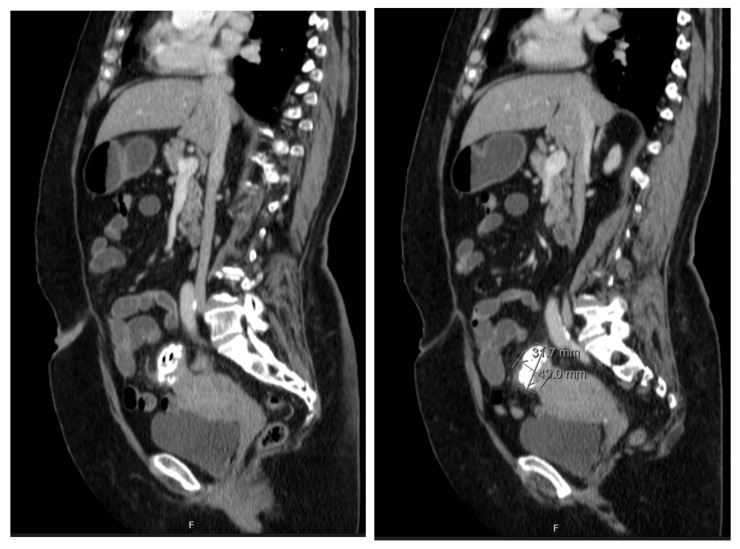
CT enterography showing a nodular, exophytic, and extensively calcified formation with two gaseous points, with dimensions of 3.4 × 3.2 × 4.3 cm, in close proximity with uterine fundus and adjacent sigmoid colon and conditioning a slight colonic retraction and surrounding fat enhancement.

The patient underwent a Computed Tomography (CT) Enterography requested by the gastroenterologist. This exam showed a mass adjacent to the uterine fundus and in continuity with the sigmoid colon wall, with a nodular and exophytic appearance, dimensions of 3.4 × 3.2 × 4.3 cm, and identifiable calcifications and gas areas and conditioning a slight colonic retraction and fat enhancement in the surrounding area (Figure 2). The blood examinations and serum tumour markers (CA 125, CEA, and CA 19.9) were unremarkable.

The differential diagnosis of colonic ulcer or uterine fistula was made. A malignancy of the myometrium (leiomyosarcoma) or other malignancies were also considered, even though less likely. 

**Figure 3:**
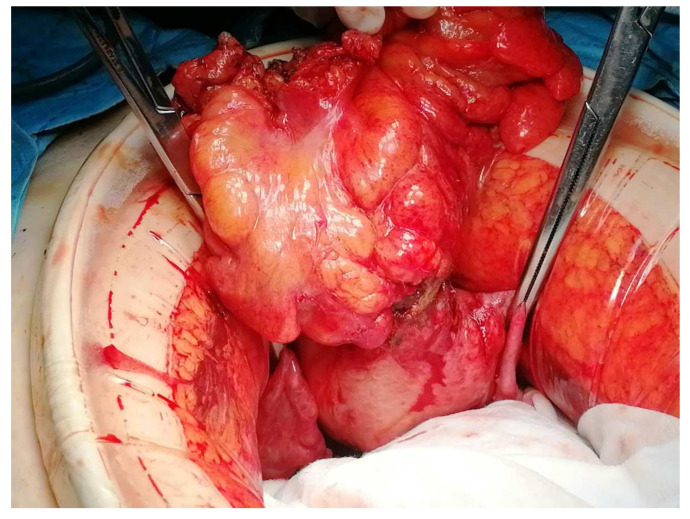
Intraoperative findings showing the sigmoid colon adherent to the serosal surface of the uterine fundus with a fistulous communication between the two viscera.

**Figure 4:**
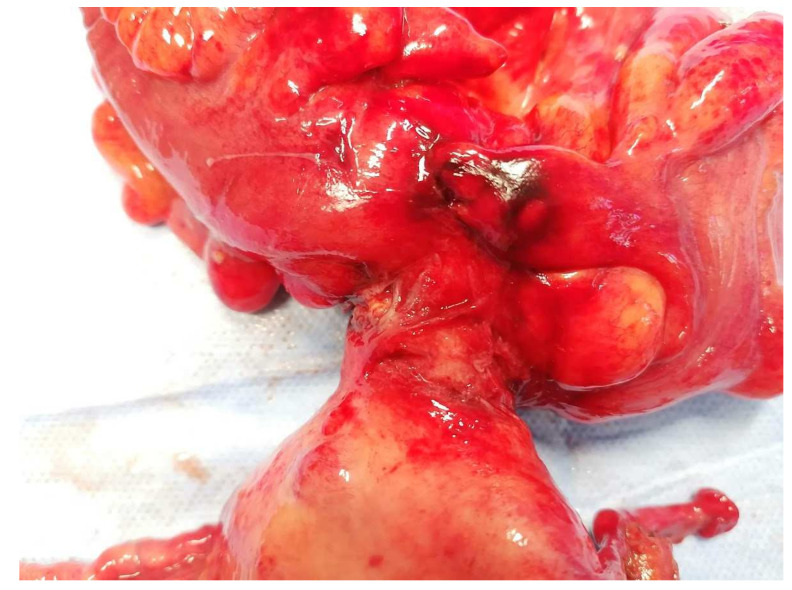
A close look at the sigmoid colon adherent to the serosal surface of the uterine fundus with a fistulous communication between the two viscera.

The treatment approach was discussed in a multidisciplinary team with the involvement of Gynaecology, Gastroenterology, General Surgery, Pathology, and Radiology. The patient underwent a programmed exploratory laparotomy via a midline incision which identified the sigmoid wall adherent to the uterine fundus with an approximately 3 cm extension; the uterus was normal in size and consistent with the small myomas identified; the adnexa were atrophic; and there were no adhesions or other abnormal findings. The procedure continued with en bloc colonic resection and primary anastomosis in the same operative time, total hysterectomy, and bilateral adnexectomy. Intraoperative frozen section examination revealed fibrous and unspecific inflammatory tissue without malignant aspects and, therefore, the procedure was finalized (Figure 3 and Figure 4). 

Post-operative recovery was uneventful, and the patient was discharged after the sixth post-operative day without complications, normal gastrointestinal habits, and no other complaints. One year after the surgery, the patient is well without complications or symptoms.

The final pathology result showed an 11 cm sigmoid colon with an irregular serous layer and adherent to uterine fundus. The microscopic examination showed a colonic diverticulum without its epithelial lining and abundant granulation tissue and fibrinous exudate extending into the uterine wall with signs of haemorrhage and chronic inflammation, which was diagnostic of a colouterine fistula of diverticular aetiology with no evidence of malignancy in the colon or the uterus. The endometrium was thin and atrophic, without malignant or atypical aspects. The uterine wall comprised three small myomas and the ovaries and tubes had no abnormalities.

A diagnosis of diverticulitis and fistulisation into the uterine cavity was made.

## 3. Discussion

Abnormal uterine bleeding is a frequent clinical sign and symptom and is reported in up to 10% of postmenopausal women, accounting for approximately two-thirds of gynaecological office visits [1]. The most common aetiology of postmenopausal abnormal uterine bleeding is endometrium atrophy due to an hypoestrogenic environment that causes endometrium epithelial microerosions and an inflammatory environment, leading to endometrium detachment and scarce bleeding [4]. Nevertheless, other causes of bleeding should be considered in this age group. The hypothesis of a uterine leiomyoma should be considered, and in these cases, it is postulated that the bleeding in the postmenopausal period occurs due to the overlining atrophic endometrium and the compressive effect in the contralateral uterine wall, both causing endometrium microerosions and inflammatory changes, similarly to the mechanisms described in the endometrium atrophy [5]. The hypothesis of an endometrium carcinoma should be considered, since it occurs in approximately 10% of postmenopausal women presenting with abnormal uterine bleeding [6]. Other malignant causes are also described, such as uterine sarcoma, but they occur rarely.

A colouterine fistula is an unusual occurrence and few cases are reported in the literature worldwide. Its rarity can make the clinical suspicion less likely and the diagnosis more difficult. It is postulated that colouterine fistula formation is not a frequent occurrence due to the thick muscular structure of the uterus that can usually provide a protective and firm barrier against an inflammatory or malignant disease, making its invasion or affection difficult. The aetiology of fistula involving the reproductive tract includes the rupture of an abscess into the adjacent viscera; malignant disease involving the gynaecologic, colon, or other pelvic organs; inflammatory bowel disease; surgery complications; and radiotherapy complications [7,8,9,10,11,12]. A colouterine fistula due to colonic chronic diverticular disease is less likely to occur.

A colonic diverticulum is a sac-like protrusion of the colonic wall through weak points in the muscular wall and, when inflammation in or adjacent to a diverticulum occurs, an episode of colonic diverticulitis occurs [13]. These inflammatory events can clinically manifest as an acute disease and/or have chronic evolution. The prevalence of this condition is higher in older patients, male sex, patients with smoking habits, and patients with elevated body mass index, and it affects the sigmoid colon more frequently [13]. The complications described in association with diverticular disease include local inflammation, abscess formation, colonic bleeding, and fistulization to adjacent organs. Moreover, fistula formation is one of the complications of diverticulitis and usually affects the sigmoid segment of the colon, most frequently involving the urinary bladder (colovesical fistula) in 65% of cases, the vagina (colovaginal fistula) in 25%, and the small bowel (coloenteric fistula) in 7% of cases. The involvement of the uterus (colouterine fistula) is described in only a small minority of cases [14].

The fistula formation of the colon and uterus occurs due to inflammatory adhesion of the colon and uterus during an episode of diverticulitis, which results in necrosis and subsequent fistula formation due to the destruction of two serosae of two epithelialized surfaces in proximity, commonly resulting in the linkage of the fundus of the uterus and the sigmoid colon [7], as we observed in this case.

The correct diagnosis and management of a colouterine fistula should include the correlation of the clinical signs and symptoms, findings of imaging, endoscopic, and other diagnostic tests, and the multidisciplinary approach of an experienced team.

The clinical presentation is variable and can vary from asymptomatic disease to a florid sepsis. In our case, presentation with abnormal uterine bleeding, without other symptoms such as abdominal pain, gastrointestinal tract changes, fever, or other significant lower gastrointestinal symptoms is very unusual. The physical examination was also unrevealing, as is described in most similar cases in the literature. Abnormal uterine bleeding, a positive faecal occult blood test, and inflammatory colonic changes seen in colonoscopy are, in fact, the main clinical signs of inflammatory or cancerous disease involving the sigmoid colon and uterus [6,8]. Together, these signs should prompt clinicians to investigate with imaging modalities, namely gynaecological ultrasound and the appropriate colonic imaging, with CT being the most commonly used.

In our case, diagnoses using imaging or endoscopy were not completely conclusive. The CT enterography findings were suggestive of diverticulitis and colouterine fistula since it showed air bubbles adjacent to the uterine cavity and the colon wall joined to the uterus. Nevertheless, definitive diagnosis was limited because CT findings cannot accurately distinguish inflammatory from neoplastic disease and may not be conclusive when distinguishing a fistula tract from complicated diverticulitis.

Diverticular fistulas do not generally close spontaneously and, therefore, it is appropriate to select a surgical approach to successfully manage these patients. The choice of en bloc resection and primary anastomosis was appropriate in our case, considering patient conditions, intraoperative findings, viability of colonic tissue, and team expertise, allowing complete treatment and resolution. The surgical approach consisted of identifying the affected colonic segment and its resection, as well as uterus and adnexa removal. In fact, malignant aetiology was not completely excluded preoperatively; the colon could not be completely separated from the uterine fundus due to chronic inflammation and the uterus and adnexa were easily removed, so there was no need to preserve the uterus and adnexa and close the fistulous opening, avoiding potential future complications such as infections and future need for uterine drainage. The discussion in a multidisciplinary team involving Gynaecology, General Surgery, Gastroenterology, Radiology, and Pathology was successful since it allowed the discussion of multiple views and insights, the decision of cooperation in a one-time procedure, the joint approach in post-operative management, and the complete resolution the patient’s condition.

Furthermore, the prognosis for a colouterine fistula secondary to diverticulitis is excellent after surgery [7,9], as was observed in our case.

## 4. Conclusions

Abnormal uterine bleeding is a common symptom and an important medical problem. In postmenopausal women, it should prompt further investigation mainly to rule out the endometrial malignancies that are prevalent in this age group. Nevertheless, other less common causes should be suspected and, therefore, the diagnostic and therapeutic approach should be adapted.

A colouterine fistula is a rare cause of abnormal uterine bleeding. The aetiology related to chronic diverticular disease is highly infrequent and its accurate diagnosis may be challenging and difficult, requiring clinical, imagiological, and intraoperative correlation of findings. This case was remarkable for its unusual presentation with postmenopausal abnormal uterine bleeding, absent gastrointestinal tract symptoms, routine positive faecal occult blood test, and abnormal findings in routine colonoscopy. These cases should also raise the possibility of a malignancy presenting similarly, and therefore teams should be aware of the need to adapt the diagnostic and surgical approach accordingly. This case was successfully managed due to a multidisciplinary team involving multiple specialities, including gynaecology and general surgery, and guidance by patient factors.

## Data Availability

No new data were created.
